# GP2: The Global Parkinson's Genetics Program

**DOI:** 10.1002/mds.28494

**Published:** 2021-01-29

**Authors:** The Global Parkinson's Genetics Program

**Keywords:** Parkinson's disease, genetics, genome‐wide association, mutation

Fundamentally, the identification of causes and contributors of disease represents the first step in an etiology‐based understanding of disease. This, in turn, is a required step in the development of therapeutics targeting the underlying disease process. Parkinson's disease (PD) is believed to be a complex disorder, with disease liability driven in part by genetics. Current heritability estimates suggest that common genetic variability contributes ~22% of the disease liability in an average patient.^1^ Although this is believed to be an underestimate, it is also likely that there are nongenetic influences, such as environmental exposure or stochastic events. Genetics, however, has shown itself to be a robust, tractable, and reliable method to understand disease biology. Genetic understanding serves as a foundation for succeeding functional studies and as a central component of efforts to predict disease risk, onset, and progression and to understand disease mechanisms in individual patients. Without a reliable and complete foundation of genetic understanding, we limit our ability to develop and deploy treatments.

A large number of risk loci and causative mutations for PD have been identified; however, it is clear that the majority of genetic risk remains to be found.^1^, ^2^ Although much can be done with existing knowledge, moving forward now to expand our genetic understanding will be the foundation that will support the development of a complete view of this network, providing an array of potential therapeutic opportunities. Increasing genetic information can only serve to improve our efforts to treat disease.

Notably, our understanding of the genetic basis of PD has thus far largely been centered on research in individuals of northern European ancestry. Although some genetic discoveries have been made outside these populations, this work is the exception rather than the norm and generally focuses on the identification of rare mutations; little has been done in the identification of more common genes or genetic risk discovery.^3^, ^4^ Thus, we do not know if our current understanding is generalizable to the rest of the world and how the basis of disease varies across populations. Although it is tempting to argue that the genetic basis for PD will generally be the same across populations, we know that differences in genetic risk exist, and furthermore, there is evidence to suggest that genetic forms of disease can present differently across populations.^3^, ^5^, ^6^, ^7^, ^8^ This fundamental limitation of current research creates an inequitable situation for patients.

To facilitate the rapid expansion of our understanding of the genetic architecture of PD, both in terms of the depth and global context of this knowledge, we have created the Global Parkinson's Genetics Program (GP2; www.gp2.org). GP2 is the first supported resource project of the Aligning Science Across Parkinson's (ASAP) initiative, an audacious effort supporting PD research.^9^ GP2 is geared toward creating a worldwide collaborative effort that will first dramatically accelerate the identification of genetic contributors to disease and second establish a network of researchers who can best leverage this understanding to research, diagnose, and treat PD worldwide. Here we describe our mission, the path we have proposed to achieve this, and the core principles of data democratization, transparency, and diversity.

## 1 Mission and Underlying Principles

**FIG. 1:**
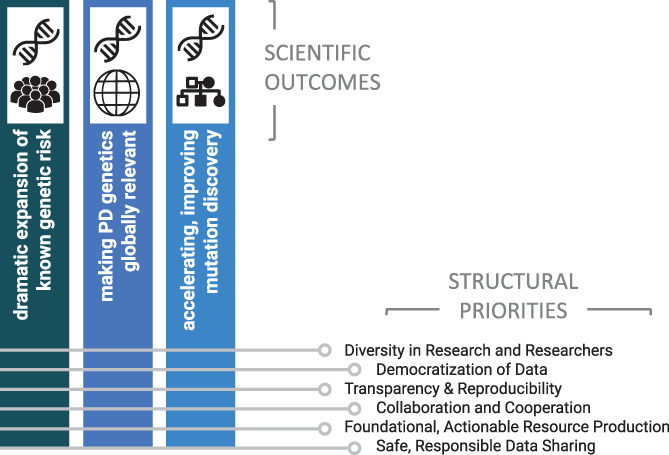
The scientific outcomes and underlying structural priorities of GP2. [Color figure can be viewed at wileyonlinelibrary.com]

The mission of GP2 is to drive transformational progress in our understanding of the genetic architecture of PD and to serve as a useful and actionable resource for the research and therapeutic development community. To fully realize this mission, GP2 will need to engage and mobilize a worldwide community of researchers and participants, generate and analyze genetic data on an extremely large scale, create an infrastructure that removes obstacles to data access, and make data and results accessible and useful to the broader community (Fig. 1).

There are several underlying principles of GP2 that we believe are central to the overall success of our work and the continued success of the research community.

1. Diversification: We will leverage the power of diversity across each dimension and in both researchers and participants.
2. Democratization: We will ensure that the data, their use, compute resources, and results are not only available but also usefully accessible to the broader research community. We will democratize GP2 and the use of GP2 data through training for contributing sites to create clinical and data‐analytical expertise locally and regionally, growing our own and collaborators’ capabilities.
3. Transparency and reproducibility: The collection, data generation, data cleaning, and analysis will be performed in an open manner. Methods, data, code, and results will be available to the research community to facilitate reproducibility, reduce redundancy, and foster community involvement in and improvement of approaches.
4. Collaboration and cooperation: We will promote a high degree of collaboration and cooperation across a global community. To be effective, this must be centered on a shared vision, collective opportunities, and responsibilities, ensuring that each member has a voice in the organization.
5. Foundational, actionable resource generation: The data and results we produce will form the foundation of a wealth of scientific and clinical research; therefore, they must be both easily available and in a form that is useful for and interpretable by the wider research community.
6. Safe, responsible data sharing: Data sharing with the research community is key; however, this must be done in a manner that ensures participant privacy and is in line with local regulations. To do this, GP2 has a working group of experts examining compliance at the level of the contributing institution and country.

## 2 Deliverables and Path

Broadly, there are 2 scientific arms to GP2, one centered in genetically complex, typical PD and the other in monogenic disease. Over the initial 5‐year span of the GP2 program, our path will lead us to a dramatic increase in the number of known genes, disease‐causing mutations, and risk loci for both rare monogenic and typical complex PD. Furthermore, this work will be extended, for the first time at scale, to underrepresented populations from around the world. We will generate dense genetic data in more than 150,000 participants, using a genotyping array specifically designed for this purpose. We will also generate whole‐genome sequence data from more than 10,000 individuals to determine the genetic cause in as yet unsolved monogenic cases and to generate much needed reference data sets. Furthermore, we will use long‐read DNA sequencing to support the analysis of structural and repeat variability that is relatively resistant to interrogation using traditional genome sequencing methods. For the most part, GP2 will use existing or ongoing patient and cohort collections and will work with established PD consortia; however, in some instances GP2 will support the collection of additional patients, particularly from underserved communities.

**FIG. 2:**
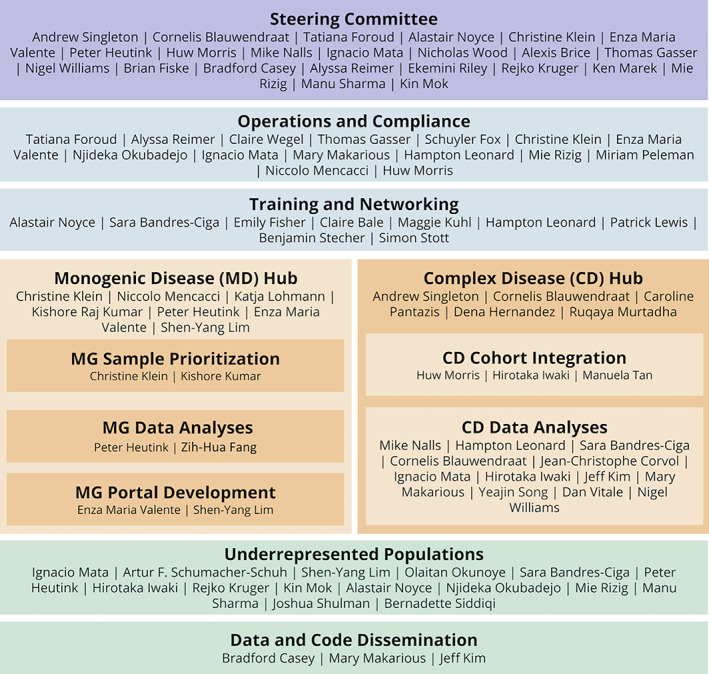
Organizational structure of GP2. [Color figure can be viewed at wileyonlinelibrary.com]

To ensure a functional and efficient structure, we created a series of working groups and hubs that center on achieving specific aims and priorities within GP2. Although these groups have clear aims and deliverables, they function as a continuum with shared members (Fig. 2).

### 2.1 Complex Disease Genetics

The role of this group is to explore the genetic basis of typical, apparently sporadic PD. The foundation of this work will be the genotyping of 150,000 participants using an array designed by us specifically for this purpose. The Neuro Booster Array is centered on the backbone of the global diversity array (GDA; 1.8+ million variants; https://www.illumina.com/products/by-type/microarray-kits/infinium-global-diversity.html) but also includes more than 95,000 custom content variants that include neurological disease‐oriented content and population‐specific boosters (article in preparation; https://github.com/GP2code/Neuro_Booster_Array). Broadly, we expect to generate data on ~100,000 northern European ancestry individuals and more than 50,000 subjects from underrepresented populations from around the world. We have established collaborations to collect and assess cases of Black American, East Asian, African, Indian, Caribbean and Central/South American provenance. Our estimates of sample numbers are driven by a combination of resource availability and pragmatism. However, we hope to significantly increase the collection and genetic analysis of patients from underrepresented groups well beyond these numbers, so 50,000 can be considered a lower bound. This will depend on the availability of additional subjects and how these cohorts are able to align with the GP2 general principles around data access and use. It is also worth noting that the majority of GP2 genotyping will be centered on persons with PD because abundant data on controls already exist; however, some of our genetic resources will be centered on generating genetic data in controls, primarily for populations in which no such data exist or for whom there are valuable PD related data in these particular control samples (eg, biomarker data).

Data from underrepresented populations will be generated from a variety of sources. GP2 has already initiated partnerships with academic research centers in the United States to improve representation of Black Americans within the project. Samples and data are being collected from East Asia by the International Parkinson's Disease Genomics Consortium (IPDGC) East Asia group, with efforts ongoing in Taiwan, Japan, South Korea, and China. Likewise, IPDGC Africa has initiated collaborations across Africa beginning with patients from Nigeria, Egypt, Ethiopia, Ghana, Mali, Tanzania, Senegal, South Africa, Sudan, and Zambia. The Genetic Epidemiology of Parkinson's disease (GEoPD) Consortium has developed collaborations across underrepresented populations from North and sub‐Saharan Africa, Australia, and Asia. The Luxembourgish‐German Indian Alliance on Neurodegenerative Diseases and Therapeutics (Lux‐GIANT) has formed a collaborative group to investigate PD patients across India.^10^ Last, the Latin American Research Consortium on the Genetics of Parkinson's Disease (LARGE‐PD) group is a fully active collaborative group collecting and investigating patients from Argentina, Brazil, Chile, Costa Rica, Colombia, Ecuador, Honduras, Mexico, Peru, Puerto Rico, Uruguay, and the West Indies.^11^ As GP2 continues, there will be room to expand to other countries and populations underserved in our current research.

These data, collectively, will afford the opportunity to rapidly detect novel genetic risk for PD. Critically, the availability of similar data across ancestral groups will allow an assessment of the varied genetic contribution in different ancestries, including the identification of population‐specific loci, an understanding of the differences in the heritable component of disease between groups, and the generation of population‐specific genetic risk profiles. Notably, these data collectively provide the opportunity to refine association signals, with transethnic fine‐mapping.

A crucial step will be integration of clinical phenotype data. We know that there are diverse outcomes of PD including the rate of motor and cognitive deterioration and medication side effects such as levodopa‐induced dyskinesias.^12^ We believe that these will be in part genetically determined^13^, ^14^ and that understanding the associated genes and pathways will lead to new biological insights and importantly personalized treatments. A barrier to this is the harmonization of data, which is a major goal of the cohort integration group.

### 2.2 Monogenic Disease Genetics

Although modern genetic methods provide tools for the rapid discovery of rare causal or high‐risk mutations, several barriers exist that limit the efficiency of identifying novel causes of disease. First, multiplex families are overall rare and dispersed. Second, the generation and analysis of genetic data are specialized and expensive. Third, existing genetic data are not harmonized and often not available for sharing. And, fourth, penetrance is reduced in dominantly inherited forms and, although high in recessive forms, age dependent. The latter results in frequent absence of the most prominent red flag for the occurrence of a monogenic form of PD, that is, positive family history, so that a significant proportion of patients miss out on genetic testing and research because they are not deemed good candidates. Collectively, this means that finding segregating mutations, or mutations in the same putative novel gene, is difficult; this, in turn, has resulted in the publication of a growing number of potentially disease‐associated mutations that are preliminary and can be quite misleading or confusing to the field. Importantly, and unlike findings from complex genetic studies of PD, these putative new monogenic causes are often readily implemented in PD gene panels for diagnostic testing by genetic testing companies, posing an additional challenge to patients, unaffected carriers, genetic counselors, and physicians in terms of interpretation of the ensuing test results.

The monogenic disease arm of GP2 aims to address these obstacles and thereby create an efficient infrastructure to accelerate the identification of novel genetic causes of apparently monogenic PD. Leveraging the above‐described global network of researchers contributing patient samples to Neuro Booster Array genotyping and including already‐existing resources from the monogenic field, such as IPDGC,^15^ the GEoPD, and the Michael J. Fox Foundation Global Genetic Parkinson's Disease Study Group,^16^ the monogenic arm will collect >5000 patients and families in whom a monogenic cause may be suspected. Particular emphasis will be placed on families from underrepresented populations. All currently known PD genes have been found in various populations around the globe, however, some occur at highly variable and population‐specific frequencies, the most striking examples being the p.G2019S mutation in the *LRRK2* gene^17^ and *GBA* mutations including p.N370S^18^ and p.K198E.^19^ In addition, it is conceivable that population‐specific hereditary forms of PD may exist, as exemplified by X‐linked dystonia‐parkinsonism, a condition exclusively present in patients of Filipino ancestry,^20^ for which the underlying genetic cause has been identified as well as genetic age‐at‐onset modifiers.^21^, ^22^, ^23^ This condition has served as an important model for basal ganglia disease.^24^, ^25^

The monogenic arm will collect families, singleton cases, and patient‐parent trios and prioritize these for whole genome sequency or long‐read DNA sequencing based on a number of different criteria: family history and availability of samples from several affected (and unaffected) family members, age at onset, ethnicity (with a focus on underrepresented populations), and level of available genetic prescreening. Importantly, all patients enrolled in the monogenic arm of the project will also undergo Neuro Booster Array genotyping, which, based on its PD‐related custom content, will result in the identification of a sizable number of patients with mutations in known PD genes. Assuming that an average of ~10%–15% of all PD patients carry a mutation in a known PD gene (3%–5%) or a high‐risk variant in *GBA* (8%–10%),^26^ we estimate a total of ~15,000 monogenic or high‐risk variant carriers to be detected in the total GP2 sample set. It is at this important interface, where the complex genetic and monogenic arms will interact most closely: although the complex genetics arm will identify carriers of known PD‐causing mutations that can then be enrolled in various additional investigations, such as genetic modifier studies of age at onset, the monogenic hub will contact all submitters of patients to the monogenic hub to also enroll patient and control cohorts into the complex genetics hub. This interplay will create unprecedented opportunities not only for the discovery of novel genetic causes of PD but also for a better understanding of the known genetic forms of PD.

## 3 The Democratization of Data Resources

Over the last 15 years, the genetics field has made great strides toward making data available to the research community. However, barriers still exist. Data are typically highly dispersed across silos/portals, there is often considerable administrative burden for data access especially when accessing multiple data sets, and data use agreements can be restrictive. In addition, the analytical expertise to interpret results or analyze data can be high, and the cost of data analysis plus curation can be prohibitive. A key stated outcome of GP2 is the generation of data and analyses that can be readily accessed and interpreted by the broad research community. To achieve this, we are taking several steps to consolidate data in one place and provide all analysis scripts with the necessary context in GitHub (https://github.com/GP2code), a public domain.

We will place as much information as timely and responsibly possible in the public domain, without requiring an extensive data‐use agreement. Although the protection of participant's data must be a priority, a large number of analyses only require summary results, and many of these can be shared publicly. Deidentified participant‐level data will be stored securely; however, we will only require a single data‐use agreement to access these cohorts, streamlining the data access process. An essential step in the onboarding process for collaborating cohorts is the consent and regulatory review process performed by the Operations and Compliance Working Group. This group works with investigators and institutional officials to surface any cohort‐specific requirements for international data sharing. Our ability to comply with these regulations while maintaining our philosophy of data accessibility and open science are taken into account when deciding if we would like to move forward with integrating the cohort into GP2.

We will follow a model in which researchers analyze data in place, rather than downloading data to local computers. This approach offers several advantages. It means that we can reduce redundancies by capturing standard quality control and analyses and sharing these as a common path. It means we can create collaborative and training opportunities and resources by working across a common, shared workspace. In GP2, we can be both standardized and transparent in our analyses, which can be easily shared with the research community, allowing independent testing, additive analyses, and crowd‐sourced improvements to workflows. This approach democratizes the data, tools, and infrastructure, allowing individuals with the skill set but without the computing resources to access and analyze these data. The model of analysts coming to the data, rather than vice versa, is one being increasingly used or promoted by major national and global genetics and genomics initiatives such as the International Complex Disease Alliance (see white paper at https://www.icda.bio/) and the AllOfUs initiative (https://allofus.nih.gov/). To achieve these aims, our current model is centered around Terra (https://terra.bio/) with other platform options possible soon.

Terra is a cloud‐based, scalable platform developed and actively maintained by the Broad Institute (https://www.broadinstitute.org/) specifically for biomedical research. Terra supports direct access and analysis of data, eliminating the need for data download. Terra workflows allow researchers to perform whole analytical pipelines, such as aligning sequence data per sample and joint calling across populations and, combined with notebooks, follow an intuitive structure and interactive analysis. Thus, by sharing notebooks and workspaces Terra makes collaboration easy and transparent. This allows researchers to easily use and reproduce workflows, reducing redundancy and maximizing transparency.

## 4 Training and Networking

It is not enough to just make data available to the wider PD research community. A key part of GP2 is to develop training opportunities that will benefit clinicians and researchers around the world so that they may pursue their own questions using GP2 data.

We recognize that a significant barrier to those wishing to work with genetic data is expertise. These training opportunities aim to establish broad, foundation‐level knowledge in genetics, bioinformatics, medical statistics, and molecular biology through a suite of new web‐based materials. Our program of web‐based training has been brought into sharp focus by the COVID‐19 pandemic, and we have accelerated our efforts to deliver educational material despite travel restrictions. This has had the benefit of making these training opportunities more readily accessible to investigators from around the world. We also aim to support clinical training opportunities in regions that would benefit most from this. Our hope is that these resources will increase the efficiency of and participation in GP2, particularly for those where such training is typically difficult to access.

For individuals who demonstrate exceptional drive and talent, whatever their background, we will support a range of individual formal training opportunities in the form of taught courses (such as master's degrees), customized training visits at centers of excellence, and full PhD studentships. We are committed to serving the needs of clinicians and researchers who have been underrepresented so far in research, and we will enable research training opportunities and build sustainable partnerships through a network of GP2 collaborators.

## 5 Promoting Diversity

We believe that a critical component of expanding research to underserved and underrepresented populations is ensuring ownership of local studies by local researchers, an active voice and role in the global study, and training, development, and other infrastructure support for local researchers to build on their expertise.

As noted above, we aim to support the development of local, clinical, and analytical expertise. Further, all data are returned to contributing investigators immediately on completion of harmonization and quality control. Using a shared compute space, the individual investigator can access the processing and quality control workspaces that have been used to generate these data. Local investigators can also use the skills transferred from training to perform collaborative analysis in GP2‐supported cloud computing, removing the barrier of providing their own infrastructure. GP2 also aims to provide several layers of analytical support beyond training. We have a data analysis core that not only serves to perform study‐wide analyses but also to support members in the form of direct analytical services, partners in one‐on‐one training, and by providing a data concierge service.

## 6 Uses of These Data

The work planned for GP2 will provide foundational data and fundamental insights for PD research. We believe that this work touches almost every aspect of the path from basic biological understanding to therapeutic deployment. Most obviously, the work from GP2 will lead to a significant increase in the proportion of the known heritable component of disease, identifying novel risk factors, mutations, and disease‐linked genes.^2^ This will improve the genetic component of multimodal risk prediction models and will provide us with greater power to determine whether genetic subtypes of the common disease are present.

Based on prior experience, the expansion of efforts into traditionally underrepresented populations will also lead to the identification of new loci that are either absent or of weaker effect in the northern European population.^3^, ^5^, ^6^ These data will allow us to determine whether heritability differs substantially across ancestral groups and to construct ancestry‐specific risk profiles. It will provide insight into the genetic basis of differences in disease presentation and course, as well as highlighting which groups may be particularly suited to a distinct etiologic‐based therapeutic approach. Further, these data allow transethnic fine‐mapping to be used to reduce the critical intervals in which functional risk alleles reside and to identify the functional effector gene.

Beyond risk, GP2 provides the opportunity to examine the genetic basis of variability in disease. Age at onset, progression, and the range of symptoms and comorbidities are each amenable to genetic discovery. Perhaps more importantly, this work paves the way for the creation of individual risk profiles for onset and trajectories of disease.

Combining complex genetic approaches with data produced in monogenic cases also allows the identification of genetic modifiers of disease penetrance.^26^, ^27^ Furthermore, these data suggest that such work may reveal differential effects of the same risk loci in primarily monogenic and complex disease, a potential functional basis for etiologic differences or at least different etiologic weights between patients. This area is of stated interest to our colleagues in industry who are looking for potential modifiers of the disease process.

### 6.1 Broad Applicability

There are a wide variety of opportunities provided by the GP2 study that are not part of its most immediate scope, and there is significant potential for additional work made possible through the generation of GP2 data. Our partnership with the PPMI study (https://www.ppmi-info.org/) enables the use of genetics in biomarker development work, integrating genetics in multimodal disease predictions, an examination of the influence of ancestry on biomarkers, and the use of genetics to improve biomarker readouts by adjusting for genetic influence unrelated to disease.

Like PPMI, many studies have collected additional data that can be analyzed with genetics. The availability of imaging and neuropathology data provides an opportunity to understand the genetic basis of variance in these measures and may offer insights on disease subtyping. Combining data from largely unbiased methods such as transcriptomics, epigenomics, metabolomics, and proteomics with genetics provides the opportunity to map quantitative trait loci in both health and disease, an approach that has been used to understand basic etiology and improve biomarker accuracy.

The sponsorship of GP2 in collecting samples from underrepresented populations also opens up the opportunity to perform ancillary studies in these groups. At the most basic level this may include simple phenotypic characterization of patients from countries or ancestral groups in which little has previously been done. It can also include work on attitudes and perspectives on disease and the use of genetics.

We believe these opportunities and myriad others in the spaces of environment, epidemiology, epigenetics, and beyond can be prioritized for further research. Our aim in GP2 is not to create an organization that tackles each of these, but rather to create a structure around which such studies can be organized. Our hope is that individual investigators within the GP2 network will propose new lines of investigation and form alliances and collaborations with other investigators to explore these questions. GP2 and our collective data and structure can serve as an initiator for such work.

### 6.2 Cellular Context and Function

The identification of novel PD‐linked gene mutations and loci will add insight into the underlying basis and biology of disease. Integrating these results with other genomic data can provide compelling evidence on the cellular context of genetic risk, a key step in ensuring the relevance of modeling efforts.^1^, ^28^, ^29^ Likewise, the more genetic information we possess, the more complete is the picture we can construct of the pathogenesis of disease. ASAP has supported a series of ambitious projects within the Collaborative Research Network (https://parkinsonsroadmap.org/research-network/) with a major focus on work to determine the biology of PD‐associated genetics. GP2 will be a foundational resource for these studies. In the relatively short time that we have reliably been able to identify genetic risk factors at scale, the translation to mechanistic understanding has been challenging. However, recent advances, particularly in the space of single‐cell genomics, promise to elucidate the cellular context, effector gene, and immediate biological effect. Once defined, the cellular context and disease‐relevant effect on expression can be used in traditional reductionist and systems approaches to define disease pathways and networks. Regardless of the path taken, a fundamental understanding of the breadth and depth of genetic influence in PD will enhance our ability to understand the disease process.

### 6.3 Informing Trial Design

GP2's work can afford us insights into the idea of mechanistic subsetting of disease, whether there are many distinct networks involved in the disease process and the distribution of these mechanisms across a typical PD population. Critically, if we find enriched or divergent mechanisms in subsets of patients, it will be important to match patients with mechanistic personalized therapeutics. This is, in essence, an extension of the rational idea of testing (for example) *LRRK2* kinase inhibitors initially in *LRRK2* mutation‐positive cases. Further, GP2 will improve prediction of disease risk and progression. This information will serve to facilitate improved clinical trial design, supporting trials in early (even preclinical) patients and adjusting outcomes based on individualized predictions of progression.

## 7 The Future

As discussed above, there are a large number of potential projects and opportunities associated with the GP2 data and collaborative framework. We are clearly at the beginning of our journey, but it is certainly difficult to resist the temptation to speculate on what priorities and opportunities may emerge as we continue.

The results of our current work inform the course forward. The efforts of the monogenic group are already providing a clearer understanding of what is left to be found. Can we identify modifiers to monogenic disease? How close are we to identifying the majority of these factors? What critical features remain to determine which mutation carriers will express disease, and when? Likewise, data from the complex genetics group will address similar questions. We will be better able to understand when we have reached biological saturation in common risk loci — the point at which newly identified loci are failing to add new insight into disease biology — perhaps an appropriate stopping point for genome‐wide association studies. We will also have a good idea of whether scaling up our sequencing efforts in complex disease makes sense — do we see significant additional discoveries or resolution using this method over genotyping?

At the simplest level, scale is immediately compelling, and it is apparent even now that it would be particularly important to scale up our work in underrepresented groups. We know our current efforts in underrepresented groups represent a significant commitment and a major step forward for the field. However, they are just a start. It will be important to capitalize on this momentum and continue to invest in research within and by underserved communities so that we can realize the potential of this work.

Last, we should also consider the next immediate steps after genetic discovery. In many ways genetics has evolved into a commodity. We have a clear path forward and broad consensus on the road to take. This is also becoming true for the next immediate steps to translate from genetic maps to mechanism, and there may be some advantage to GP2 tackling some of these problems, particularly around the creation of resources to make this translation efficient. However, regardless of how we get to these next steps, we believe that GP2 will serve as the starting point on this journey and a foundation for the global PD research community. We are excited about the progress we have made so far and thrilled to be making this journey with our partners.

## 8 Author Roles

Members of GP2 (contributors) drafted and made critical revisions to this article

## Appendix A.

Global Parkinson's Genetics Program Members

**Table:**

Name	Institute
Ai Huey Tan	Division of Neurology, Department of Medicine and the Mah Pooi Soo & Tan Chin Nam Centre for Parkinson's & Related Disorders, Faculty of Medicine, University of Malaya, Kuala Lumpur, Malaysia
Alastair Noyce	Preventive Neurology Unit, Wolfson Institute of Preventive Medicine, Barts and the London School of Medicine and Dentistry, Queen Mary University, London, United Kingdom
Alejandro Martinez Carrasco	Department of Clinical and Movement Neurosciences, UCL Queen Square Institute of Neurology, London, UK
Alexis Brice	Institute for Brain and Spinal Cord, Paris, France
Alyssa Reimer	The Michael J. Fox Foundation for Parkinson's Research, New York, NY, USA
Anastasia Illarionova	German Center for Neurodegenerative Diseases (DZNE), Tuebingen, Germany
Andrew Singleton	Molecular Genetics Section, Laboratory of Neurogenetics, National Institute on Aging, National Institutes of Health, Bethesda, MD
Artur Schumacher‐Schuh	Universidade Federal do Rio Grande do Sul, Hospital de Clínicas de Porto Alegre, Porto Alegre, Brazil
Benjamin Stecher	Independent patient advocate and consultant
Bernadette Siddiqi	The Michael J. Fox Foundation for Parkinson's Research, New York, NY
Bradford Casey	The Michael J. Fox Foundation for Parkinson's Research, New York, NY
Brian Fiske	The Michael J. Fox Foundation for Parkinson's Research, New York, NY
Caroline Pantazis	Laboratory of Neurogenetics, National Institute on Aging, National Institutes of Health, Bethesda, MD
Christine Klein	Institute of Neurogenetics, University of Luebeck, Luebeck, Germany
Claire Bale	Parkinsons UK, London, UK
Claire Wegel	Department of Medical and Molecular Genetics, Indiana University School of Medicine, Indianapolis, IN
Cornelis Blauwendraat	Laboratory of Neurogenetics, National Institute on Aging, National Institutes of Health, Bethesda, MD
Dan Vitale	Data Tecnica International, Glen Echo, MD
Dena Hernandez	Laboratory of Neurogenetics, National Institute on Aging, National Institutes of Health, Bethesda, MD
Ekemini Riley	Aligning Science Across Parkinson's, Chevy Chase, MD
Emily Fisher	Preventive Neurology Unit, Wolfson Institute of Preventive Medicine, Barts and the London School of Medicine and Dentistry, Queen Mary University, London, UK
Enza Maria Valente	Department of Molecular Medicine, University of Pavia and IRCCS Mondino Foundation, Pavia, Italy
Eva Juliane Vollstedt	Institute of Neurogenetics, University of Luebeck, Luebeck, Germany
Faraz Faghri	Data Tecnica International, Glen Echo, MD
Hampton Leonard	Data Tecnica International, Glen Echo, MD
Hirotaka Iwaki	Data Tecnica International, Glen Echo, MD
Huw Morris	Department of Clinical and Movement Neurosciences, UCL Queen Square Institute of Neurology, London, UK
Ignacio Juan Keller Sarmiento	Northwestern University, Feinberg School of Medicine, Department of Neurology, Chicago, IL
Ignacio Mata	Genomic Medicine Institute, Cleveland Clinic & Molecular Medicine, Cleveland Clinic Lerner College of Medicine of Case Western Reserve University, Cleveland, OH
Jeff Kim	Laboratory of Neurogenetics, National Institute on Aging, National Institutes of Health, Bethesda, MD
John Hardy	Department of Clinical and Movement Neurosciences, UCL Queen Square Institute of Neurology, London, UK
Kaileigh Murphy	The Michael J. Fox Foundation for Parkinson's Research, New York, NY
Katja Lohmann	Institute of Neurogenetics, University of Luebeck, Luebeck, Germany
Ken Marek	The Michael J. Fox Foundation for Parkinson's Research, New York, NY
Kin Mok	UK Dementia Research Institute at UCL and Department of Neurodegenerative Disease, UCL Institute of Neurology, University College London, London, UK
Kishore Kumar	Molecular Medicine Laboratory, Concord Hospital, University of Sydney, Garvan Institute, Sydney, Australia
Lara Lange	Institute of Neurogenetics, University of Luebeck, Luebeck, Germany
Maggie Kuhl	The Michael J. Fox Foundation for Parkinson's Research, New York, NY
Manu Sharma	Centre for Genetic Epidemiology, Institute for Clinical Epidemiology and Applied Biometry, University of Tübingen, Tübingen, Germany
Manuela Tan	Department of Clinical and Movement Neurosciences, UCL Queen Square Institute of Neurology, London, UK
Mary Makarious	Laboratory of Neurogenetics, National Institute on Aging, National Institutes of Health, Bethesda, MD
Michelle Durborow	The Michael J. Fox Foundation for Parkinson's Research, New York, NY
Micol Avenali	Department of Brain and Behavioural Sciences, University of Pavia and IRCCS Mondino Foundation, Pavia, Italy
Mie Rizig	Department of Molecular Neuroscience, UCL Institute of Neurology and National Hospital for Neurology and Neurosurgery, London, UK
Mike Nalls	Data Tecnica International, Glen Echo, MD
Niccolo Mencacci	Northwestern University, Feinberg School of Medicine, Department of Neurology, Chicago, IL
Nicholas Wood	Department of Clinical and Movement Neurosciences, UCL Queen Square Institute of Neurology, London, UK
Nigel Williams	MRC Centre for Neuropsychiatric Genetics and Genomics, Institute of Psychological Medicine and Clinical Neurosciences, Cardiff University, Cardiff, UK
Njideka Okubadejo	Neurology Unit, Department of Medicine, College of Medicine, University of Lagos, Idi Araba, Lagos State, Nigeria
Olaitan Okunoye	Department of Movement and Clinical Neurosciences, Queen Square Institute of Neurology, University College London, London, UK
Patrick Lewis	Comparative Biomedical Sciences, Royal Veterinary College, London, UK
Peter Heutink	German Center for Neurodegenerative Diseases (DZNE), Tuebingen, Germany
Prashanth LK	Parkinson's Disease & Movement Disorders Clinic, Vikram Hospitals, Bangalore, Karnataka, India
Rejko Kruger	Luxembourg Institute of Health (LIH), Strassen, Luxembourg; Luxembourg Centre for Systems Biomedicine (LCSB), University of Luxembourg, Esch‐sur‐Alzette, Luxembourg; Centre Hospitalier de Luxembourg (CHL), Luxembourg
Roopa Rajan	All India Institute of Medical Sciences, New Delhi, India‐ 110029
Ruqaya Murtadha	Laboratory of Neurogenetics, National Institute on Aging, National Institutes of Health, Bethesda, MD
Sabina Adams	Preventive Neurology Unit, Wolfson Institute of Preventive Medicine, Barts and the London School of Medicine and Dentistry, Queen Mary University, London, UK
Sara Bandres‐Ciga	Laboratory of Neurogenetics, National Institute on Aging, National Institutes of Health, Bethesda, MD
Schuyler Fox	The Michael J. Fox Foundation for Parkinson's Research, New York, NY
Shen‐Yang Lim	Division of Neurology, Department of Medicine; and The Mah Pooi Soo & Tan Chin Nam Centre for Parkinson's & Related Disorders, Faculty of Medicine, University of Malaya, Kuala Lumpur, Malaysia
Simon Stott	Cure Parkinsons Trust, London, UK
Soraya Bardien	Division of Molecular Biology and Human Genetics, Faculty of Medicine and Health Sciences, Stellenbosch University, Cape Town, South Africa
Tatiana Foroud	Department of Medical and Molecular Genetics, Indiana University School of Medicine, Indianapolis, IN
Thomas Gasser	Hertie Institute for Clinical Brain Research, University of Tübingen, Tübingen, Germany
Todd Sherer	The Michael J. Fox Foundation for Parkinson's Research, New York, NY
Yeajin Song	Data Tecnica International, Glen Echo, MD
Zih‐Hua Fang	German Center for Neurodegenerative Diseases (DZNE), Tuebingen, Germany

## References

[1] Nalls MA , Blauwendraat C , Vallerga CL , et al. Identification of novel risk loci, causal insights, and heritable risk for Parkinson's disease: a meta‐analysis of genome‐wide association studies. Lancet Neurol 2019;18:1091–1102. 31701892 10.1016/S1474-4422(19)30320-5 PMC8422160

[2] Blauwendraat C , Nalls MA , Singleton AB . The genetic architecture of Parkinson's disease. Lancet Neurol 2020;19:170–178. 31521533 10.1016/S1474-4422(19)30287-X PMC8972299

[3] Foo JN , Chew EGY , Chung SJ , et al. Identification of risk loci for Parkinson disease in Asians and comparison of risk between Asians and Europeans: a genome‐wide association study. JAMA Neurol 2020;77(6):746–754. 32310270 10.1001/jamaneurol.2020.0428 PMC7171584

[4] Zhao Y , Qin L , Pan H , et al. The role of genetics in Parkinson's disease: a large cohort study in Chinese mainland population. Brain 2020;143:2220–2234. 32613234 10.1093/brain/awaa167

[5] Simón‐Sánchez J , Schulte C , Bras JM , et al. Genome‐wide association study reveals genetic risk underlying Parkinson's disease. Nat Genet 2009;41:1308–1312. 19915575 10.1038/ng.487 PMC2787725

[6] Satake W , Nakabayashi Y , Mizuta I , et al. Genome‐wide association study identifies common variants at four loci as genetic risk factors for Parkinson's disease. Nat Genet 2009;41:1303–1307. 19915576 10.1038/ng.485

[7] Gwinn‐Hardy K , Chen JY , Liu HC , et al. Spinocerebellar ataxia type 2 with parkinsonism in ethnic Chinese. Neurology 2000;55:800–805. 10993999 10.1212/wnl.55.6.800

[8] Subramony SH , Hernandez D , Adam A , et al. Ethnic differences in the expression of neurodegenerative disease: Machado‐Joseph disease in Africans and Caucasians. Mov Disord 2002;17:1068–1071. 12360561 10.1002/mds.10241

[9] Schekman R , Riley EA . Coordinating a new approach to basic research into Parkinson's disease. Elife 2019;8:e51167. 31551111 10.7554/eLife.51167 PMC6760967

[10] Rajan R , Divya KP , Kandadai RM , et al. Genetic architecture of Parkinson's disease in the Indian population: harnessing genetic diversity to address critical gaps in Parkinson's disease research. Front Neurol 2020;11:524. 32655481 10.3389/fneur.2020.00524 PMC7323575

[11] Zabetian CP , Mata IF . Latin American research consortium on the genetics of PD (LARGE‐PD). LARGE‐PD: examining the genetics of Parkinson's disease in Latin America. Mov Disord 2017;32:1330–1331. 28657124 10.1002/mds.27081

[12] Lim S‐Y , Tan AH , Ahmad‐Annuar A , et al. Parkinson's disease in the Western Pacific region. Lancet Neurol 2019;18:865–879. 31175000 10.1016/S1474-4422(19)30195-4

[13] Tan MMX , Lawton MA , Jabbari E , et al. Genome‐wide association studies of cognitive and motor progression in Parkinson's disease. Mov Disord 2020. 10.1002/mds.28342 PMC9053517 33111402

[14] Iwaki H , Blauwendraat C , Leonard HL , et al. Genomewide association study of Parkinson's disease clinical biomarkers in 12 longitudinal patients’ cohorts. Mov Disord 2019;34:1839–1850. 31505070 10.1002/mds.27845 PMC7017876

[15] International Parkinson Disease Genomics Consortium (IPDGC) . Ten years of the international Parkinson disease genomics consortium: Progress and next steps. J Parkinsons Dis 2020;10:19–30. 31815703 10.3233/JPD-191854 PMC7029327

[16] Vollstedt E‐J , Kasten M , Klein C , MJFF Global Genetic Parkinson's Disease Study Group . Using global team science to identify genetic parkinson's disease worldwide. Ann Neurol 2019;86:153–157. 31155756 10.1002/ana.25514 PMC7410260

[17] Healy DG , Falchi M , O'sullivan SS , et al. Phenotype, genotype, and worldwide genetic penetrance of LRRK2‐associated Parkinson's disease: a case‐control study. Lancet Neurol 2008;7:583–590. 18539534 10.1016/S1474-4422(08)70117-0 PMC2832754

[18] Sidransky E , Nalls MA , Aasly JO , et al. Multicenter analysis of glucocerebrosidase mutations in Parkinson's disease. N Engl J Med 2009;361:1651–1661. 19846850 10.1056/NEJMoa0901281 PMC2856322

[19] Velez‐Pardo C , Lorenzo‐Betancor O , Jimenez‐Del‐Rio M , et al. The distribution and risk effect of GBA variants in a large cohort of PD patients from Colombia and Peru. Parkinsonism Relat Disord 2019;63:204–208. 30765263 10.1016/j.parkreldis.2019.01.030 PMC7175776

[20] Pauly MG , Ruiz López M , Westenberger A , et al. Expanding data collection for the MDSGene database: X‐linked dystonia–parkinsonism as use case example. Mov Disord 2020;35(11):1933–1938. 32949450 10.1002/mds.28289

[21] Bragg DC , Mangkalaphiban K , Vaine CA , et al. Disease onset in X‐linked dystonia‐parkinsonism correlates with expansion of a hexameric repeat within an SVA retrotransposon in *TAF1* . Proc Natl Acad Sci U S A 2017;114:E11020–E11028. 29229810 10.1073/pnas.1712526114 PMC5754783

[22] Westenberger A , Reyes CJ , Saranza G , et al. A hexanucleotide repeat modifies expressivity of X‐linked dystonia parkinsonism. Ann Neurol 2019;85:812–822. 30973967 10.1002/ana.25488

[23] Aneichyk T , Hendriks WT , Yadav R , et al. Dissecting the causal mechanism of X‐linked dystonia‐parkinsonism by integrating genome and Transcriptome assembly. Cell 2018;172:897–909.e21. 29474918 10.1016/j.cell.2018.02.011 PMC5831509

[24] Weissbach A , Bäumer T , Rosales R , et al. Neurophysiological fingerprints of X‐linked dystonia‐parkinsonism: a model basal ganglia disease. Mov Disord 2015;30:873–875. 25914216 10.1002/mds.26224

[25] Hanssen H , Prasuhn J , Heldmann M , et al. Imaging gradual neurodegeneration in a basal ganglia model disease. Ann Neurol 2019;86:517–526. 31376168 10.1002/ana.25566

[26] Blauwendraat C , Reed X , Krohn L , et al. Genetic modifiers of risk and age at onset in GBA associated Parkinson's disease and Lewy body dementia. Brain 2020;143:234–248. 31755958 10.1093/brain/awz350 PMC6935749

[27] Iwaki H , Blauwendraat C , Makarious MB , et al. Penetrance of Parkinson's disease in LRRK2 p.G2019S carriers is modified by a polygenic risk score. Mov Disord 2020;35:774–780. 31958187 10.1002/mds.27974 PMC8975556

[28] Bryois J , Skene NG , Hansen TF , et al. Genetic identification of cell types underlying brain complex traits yields insights into the etiology of Parkinson's disease. Nat Genet 2020;52:482–493. 32341526 10.1038/s41588-020-0610-9 PMC7930801

[29] Agarwal D , Sandor C , Volpato V , et al. A single‐cell atlas of the human substantia nigra reveals cell‐specific pathways associated with neurological disorders. Nat Commun 2020;11:4183. 32826893 10.1038/s41467-020-17876-0 PMC7442652

